# A comprehensive overview of eco-friendly bio-fertilizers extracted from living organisms

**DOI:** 10.1007/s11356-023-30260-x

**Published:** 2023-10-18

**Authors:** Esraa E. Ammar, Hadeer A. Rady, Ahmed M. Khattab, Mohamed H. Amer, Sohila A. Mohamed, Nour I. Elodamy, Ammar AL-Farga, Ahmed A. A. Aioub

**Affiliations:** 1 Plant Ecology, Botany Department, Faculty of Science, Tanta University, Tanta, 31527 Egypt; 2 Biotechnology, Botany Department, Faculty of Science, Tanta University, Tanta, 31527 Egypt; 3 Chemistry Department, Faculty of Pharmacy, Al-Azhar University, Cairo, 11675 Egypt; 4 Department of Biochemistry, Faculty of Science, University of Jeddah, Jeddah, Saudi Arabia; 5 Plant Protection Department, Faculty of Agriculture, Zagazig University, Zagazig, 44511 Egypt

**Keywords:** Biofertlizers, Bacteria, Algae, Fungi, Monera, Plantae, Sustainable agriculture

## Abstract

Currently, sustainable agriculture involves ecofriendly techniques, which include biofertilization. Biofertilizers increase plant productivity by improving soil fertility and nutrient content. A wide range of living organisms can be applied as biofertilizers and increase soil fertility without causing pollution due to their biodegradability. The organisms can be microorganisms like bacteria, microalgae, and micro fungi or macro organisms like macroalgae, macro fungi, and higher plants. Biofertilizers extracted from living organisms or their residues will be increasingly used rather than chemical fertilizers, which cause heavy metal accumulation in soil. Biofertilizer use aims for sustainable development in agriculture by maintaining the soil. This will mitigate climate change and related impacts and will also lower many serious diseases resulting from pollution such as cancer, liver and renal failure, and immune diseases. This review is a comprehensive overview of biofertilizers extracted from a range of living organisms from the Kingdoms Monera to Plantae and included bacteria, algae, fungi, and higher plants. Organisms that play a vital role in elevating soil nutrients in a safe, cheap, and ecofriendly manner are included in the review to promote their potential commercial application.

## Introduction

The world population is projected to increase to over 10 billion people over the next 30 years. Therefore, food supply will need to rise by 60% in order to fulfil the predicted demand in 2050 (Pambuka et al. 2021, 2022; Kinge et al. 2022). Food distribution and manufacturing must be handled in a more effective and sustainable manner to prevent supply shortages. To do this, farmers are using organic fertilizers, which are made from recycled material such as manure, agricultural residues, and town sewage, including human waste (Ju et al. 2005). The misuse of pesticides and inorganic fertilizers contributed to the desertification of agricultural land and diminishing crop productivity and increased serious human disorders (Bedair et al. 2022b). So, it is necessary to explore biofertilization to obtain healthy food (Ammar 2022).

The soil ecosystem has been severely harmed by inorganic fertilizers, herbicides, and insecticides. Future crop growth will be hampered by this type of treatment on the soil. Currently, the use of natural plant bio stimulants is advocated as an inventive approach to meet the difficulties of sustainable agriculture and to assure optimal nutrient uptake, crop output, and resistance to abiotic stress (Povero et al. 2016). In order to promote the growth of high yield crops, fertilizers are necessary. Many crops on most soils require significant amounts of fundamental nutrients that plants need for healthy growth and include nitrogen (NH_4_^+^ or NO_3_), phosphorus (H_2_PO_4_), calcium (Ca^2+^), sulfur (SO_4_^2−^), magnesium (Mg^2+^), potassium (K^+^), iron (Fe^2+^ or Fe^3+^), and zinc (Zn^2+^ or Zn(OH)_2_) (White and Brown 2010).

Regardless of the amount of nutrients in the crop, bio stimulants are natural compounds or microorganisms that are administered to plants to increase their nutritional efficiency, resistance to abiotic stress, and qualitative attributes (Abdelsalam et al. 2022). As a subclass of bio stimulants, biofertilizers are microbial inoculants that can enhance the nutritional efficiency of plants by containing active or inactive formulations of advantageous microorganisms (Deepak et al. 2015; Backer et al. 2018; Alori and Babalola 2018).

Since synthetic fertilizers are of chemical origin, over application cause eutrophication, harmful to both soil and plants. Synthetic fertilizers find their way into the adjacent streams and rivers, where they contaminate water and harm fish and other aquatic animals (Sabry 2015). Plants, on the other hand, gradually and safely deliver nutrients to the soil. Many of the plants that are used to fertilize soil are initially planted as cover crops to prevent soil erosion from wind and rain. Enhancing soil fertility requires maintaining a healthy topsoil layer (Thornbro 2022), so scientists currently seek to find new technologies to manufacture new ecofriendly fertilizers from living organisms, especially microbes, which have the ability to biodegrade without any residues.

Biofertilizers enhance crop yield by about 10 to 40% and increase proteins, vital amino acids, vitamins, and nitrogen fixation (Bhardwaj et al. 2014; Shahwar et al. 2023). Biofertilizers have been suggested as a replacement for mineral fertilizers. For instance, nitrogen- and/or sulfur-fixing microorganisms have been used in biofertilizers (Demoling et al. 2007; Beneduzi et al. 2008; Singh and Reddy 2012; Heba et al. 2021) and include bacteria like *Azotobacter*, *Azospirillum*, and *Rhizobium* as well as fungi like *Aspergillus niger* and *A. tubingensis*. Genetically altered bacterial strains have been created and tested as biological fertilizers (Sharma et al. 2013). As a natural substitute for synthetic fertilizers, bacterial and fungal biofertilizers have gained popularity. However, their current application has been reduced due to their low effectiveness compared to conventional fertilizers (Nehl et al. 1997; Deepak et al. 2015; Alori and Babalola 2018). Many species of soil bacteria and fungi, which live in beneficial associations, act as ecofriendly soil fertilizers (Ammar et al. 2022; Aioub et al. 2022). Cyanobacteria such as *Nostoc* sp*.*, *Anabaena* sp., and *Oscillatoria angustissima* are potential sources of biofertilizers (Ammar et al. 2022). Cover crops contribute to the safe and gradual delivery of nutrients to the soil and stop soil erosion from wind and rain. Maintaining a sound topsoil layer is necessary for increasing soil fertility.

**Fig. 1:**
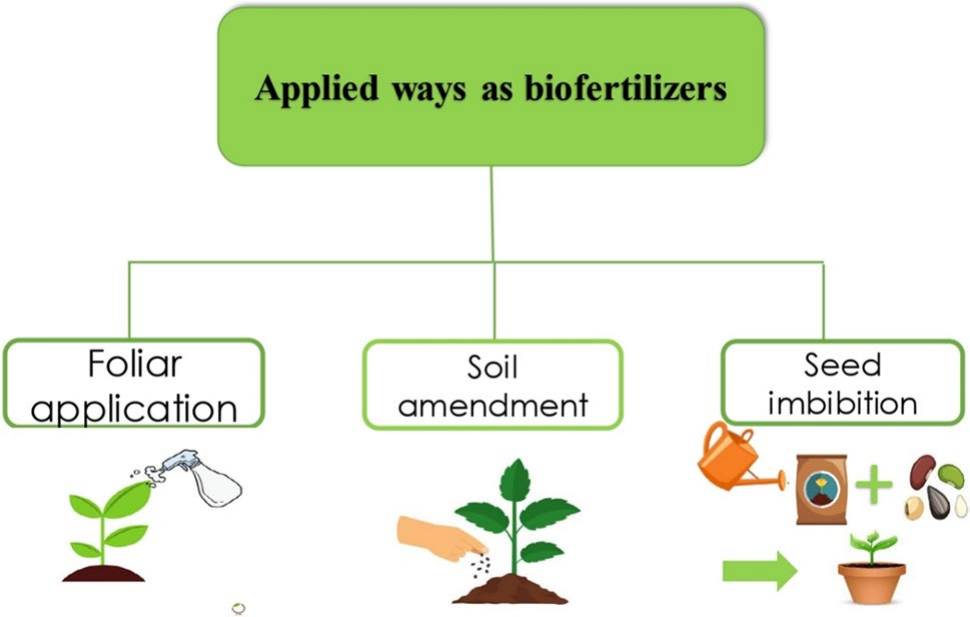
Applied ways of biofertilizer

Plants are frequently used in permaculture to increase soil fertility and residues of some plants such as *Musa paradisiaca*, *Coffea arabica*, and *Lathyrus oleraceus* can be used as soil fertilizers (Singh et al. 2013). Biofertilizers are compounds that include microorganisms and, when given to the soil, improve soil fertility and encourage plant development. In order to boost the nutrient content of the soil and thus the production, biofertilization is a sustainable agricultural practice. It has been discovered that soil microflora can increase soil fertility and boost biomass productivity and is acknowledged as an appropriate environmentally acceptable bio-based fertilizer used in agriculture to prevent pollution. Most cyanobacteria can fix nitrogen from the environment. There are three ways in which extracts of living organisms are applied as biofertilizers: foliar application (spray), soil amendment, and seed imbibition (Fig. 1).

This review summarizes the use of all types of eco-friendly biofertilizers and highlights the efficacy of biofertilizers above inorganic fertilizers. Natural biofertilizers offer high eco-economic value, reduce the risk of plant diseases, less harmful to people than inorganic fertilizers, minimize pollution, and improve soil fertility without heavy metal and residue accumulation over time due to their biodegradability (Ammar 2022).

## Bacteria as biofertilizers

Bacterial biofertilizers can increase plant growth and development through a series of various mechanisms (Kumar et al. 2022). These include the production of plant nutrients or phytohormones that can be absorbed by plants, the mobilization of soil compounds so that they are readily available for the plant to use as nutrients, and lastly, the protection of plants under stressful conditions. The later mechanisms help in counteracting the negative effects of stressor defense against plant pathogens, reducing plant diseases or death of the plant. Many plant growth-promoting rhizobacteria (PGPR) have been utilized for a long time as biofertilizers all over the world. They help to increase crop yields and soil fertility, which has the potential to help make agriculture and forestry more sustainable. The market for bacterial-based biofertilizers is expanding, and the production and application of bacterial inocula technologies are constantly being developed and improved (Garcia-Gonzalez and Sommerfeld 2016). For instance, *Azotobacter* has been extensively researched under laboratory and field conditions and has been employed as a biofertilizer for more than a century. *Azotobacter* fixes nitrogen (endophytically) in the rhizosphere and roots of rice, that promotes the growth and development of rice. The findings demonstrated that *Azotobacter* fixes atmospheric nitrogen in the rhizosphere and as an endophyte in rice and produces phytohormones and plant growth stimulants. In addition to fixing nitogen, these bacteria break down insoluble phosphate and boost plant growth (Tualar 2013; Dar et al. 2021; Daniel et al. 2022) (Tables 1 and 2).

**Table 1:** Nitrogen-fixing bacteria as biofertilizer

Bacteria	Example	Crop plant	Effect	References
Free living	*Azotobacter*	Rice	Promote plant growth	Dar et al. (2021)
Symbiotic	*Anabaena azollae*	Rice	Increases soil fertility by expanding the microbial populations in the soil	Adhikari et al. (2020); Abd El-Aal (2022)
Associative symbiotic	*Azospirillum*	Wheat, maize	Alleviation of abiotic stress	Raffi and Charyulu (2021)

**Table 2:** Phosphate solubilizing bacteria as biofertilizer

Plant growth-promoting rhizobacteria (PGPR)	Host plant	Effect	Reference
*Azotobacter chroococcum*	Wheat	Better performance with phosphate-solubilizing mutants	Nosheen et al. (2021)
*Bacillus megaterium*	Sugarcane	Yield and yield components of sugarcane growing in pots are promoted	Chungopast et al. (2021)
*Bradyrhizobium japonicum*	Soybean	Reduces negative impacts of drought stress on the growth efficiency of soybean plants	Sheteiwy et al. (2021)
*Pantoea agglomerans*	Tomato	Improved growth	Mei et al. (2021)
*Pseudomonas fluorescens*	Sweet potato	Increased yield	Santana-Fernández et al. (2021)
*Rhizobium leguminosarum*	Faba bean	Enhanced production of faba bean	Fikadu (2022)

**Fig. 2:**
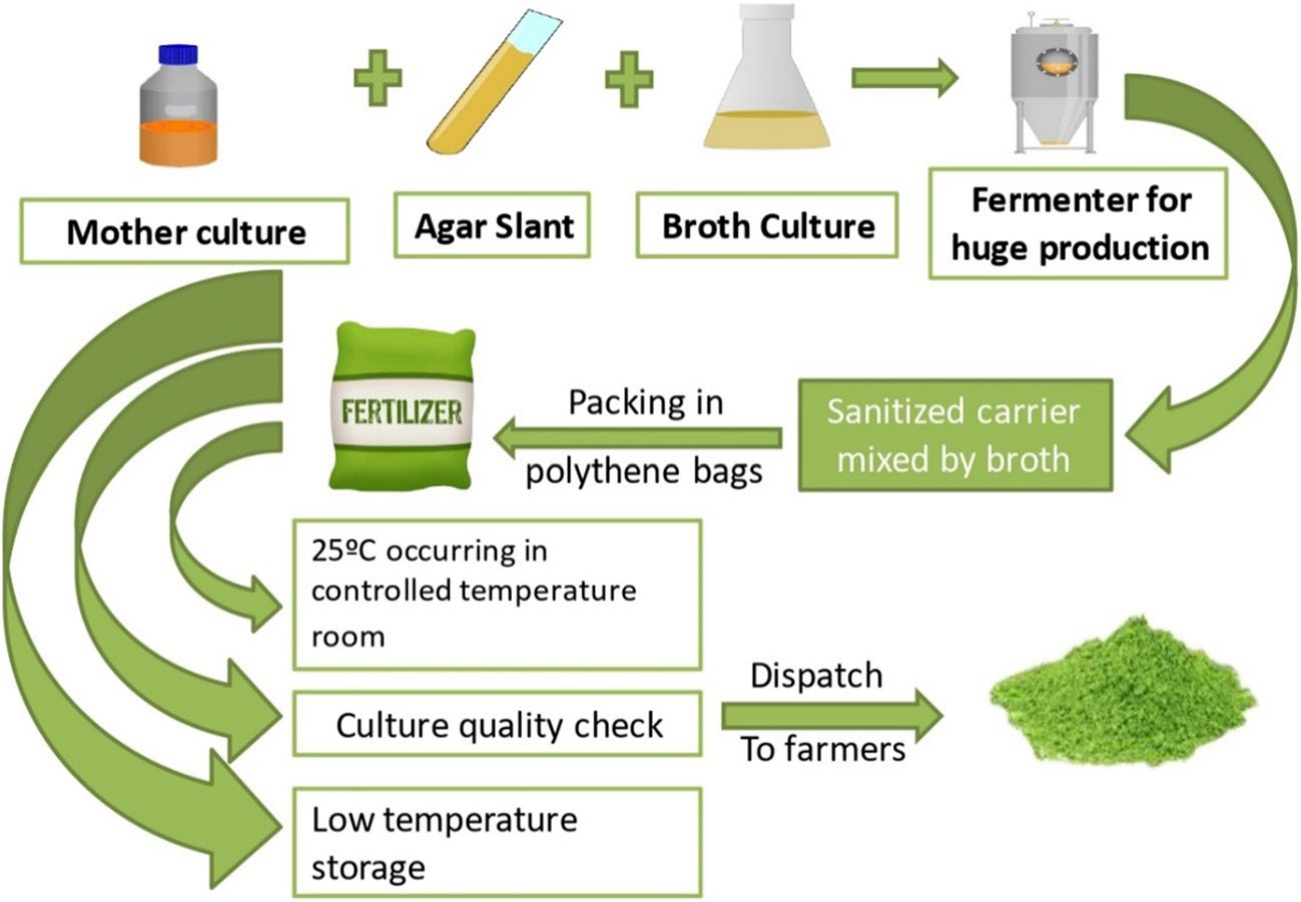
Cultivation of cyanobacteria for using in bio fertilization

On the other hand, Cyanobacteria are the oldest and most productive prokaryote group on earth and include a wide range of organisms. Cyanobacteria biomass or extracts greatly enhanced the physical and chemical properties of soil. Cyanobacteria are also widely recognized for producing biologically active compounds that are effective against plant pathogens and in the phytoremediation of industrial wastewater (Bedair et al. 2022a, b). Therefore, as natural biofertilizers, they contribute significantly to nutrient cycling, phosphorus bioavailability, N_2_-fixation, environmental protection, and disease control, and improve plant growth and production. Radiative energy is also transformed into chemical energy by cyanobacteria. Through photosynthesis, these biological systems produce oxygen. Food, energy, secondary metabolites, cosmetics, and pharmaceuticals are all products of these species. By lowering CO_2_ levels through environmentally benign large-scale growth of cyanobacteria, several high-value items can be produced. Thus in the long run, Cyanobacteria biofertilizers could take the place of chemical fertilizers (Gören-Sağlam 2021; Mishra et al. 2021; Bhuyan et al. 2022) (Tables 1 and 2). For instance, *Anabaena azollae* is a heterocystous filamentous cyanobacterium that fixes nitrogen and grows symbiotically in specific leaf cavities of the tiny eukaryotic water fern *Azolla pinnata*. *Anabaena azollae* is grown and used in synthetic medium such as BG-110. *Azollae* is a promising natural bio-source with potential uses in industry, medicine, and agriculture. Indole acetic acid, gibberellic acid, bioactive like fatty acids, polysaccharides, and phenolic compounds were isolated from *A. azollae* and reported to have microbicidal activities *in vitro* and *in vivo*. Additionally, the high nitrogenase activity of *A*. *azollae* has long been recognized as a predictor biofertilization capacity. The enhanced dehydrogenase activity and associated polysaccharide excretion increased soil fertility by expanding the microbial populations (Adhikari et al. 2020; Bao et al. 2021; Abd El-Aal 2022) (Fig. 2, Tables 1 and 2).

Similarly, *Nitrobacter* biofertilizer reduces gas emissions in rice farming, which is one of the main sources of CH_4_ emissions. It generates between 5 and 19% of the world’s overall CH_4_ emissions. Using the closed chamber method, the weekly CH_4_, CO_2_, and N_2_O gas fluxes were measured during a rice-growing season. The treatment of straw plus biofertilizers decreased the emissions of CH_4_, N_2_O, and CO_2_ by 9.2, 14.78, and 27.68%, respectively, compared to straw alone. When compared to only applying straw, the application of straw plus biofertilizers reduced global warming potential by 10.75%. The treatment with straw plus biofertilizer reduced the greenhouse gas index by 8.27% as compared to the control. The findings suggested that using biofertilizer could be environmentally friendly and promote local food security (Rani et al. 2021; Yulianingsih et al. 2021) (Tables 1 and 2). For example, *Nitrobacter* biofertilizer was researched using a two-factor factorial experiment with randomized block design with different varieties of shallots and at varying concentrations. The results demonstrated that *Nitrobacter* biofertilizer treatment produced favorable outcomes for the quantity of bulbs per cluster and dried bulbs per plot (tonnes per ha bulbs) (Saharuddin et al. 2018).

Also, *Rhizobium* is used as biofertilizer in agriculture to promote plant development as an addition to chemical fertilizers. Microorganisms that can dissolve potassium have been utilized as cyanobacteria, nitrogen-fixing bacteria, phosphate mineralizing bacteria, and a variety of crops using biofertilizers. Rhizobacteria’s ability to survive in soil is influenced by a variety of abiotic and biotic factors. The bacteria are mixed with a carrier to improve their rate of survival in soil and to increase their viability and effectiveness (Khosravi and Rahmani 2022.; Negash and Wondimu 2022) (Tables 1 and 2).

Silicate bacteria obviously play a part in the weathering of silicate minerals, but the main degrading factor appears to be the formation of acids by the microbes that live on stone. It is believed that heterotrophic bacteria and fungus secrete organic acids like oxalic, citric, or gluconic acids, which are more significant weathering agents. The effectiveness of two strains of *B*. *circulans* and one strain of *Arthrobacter tumescens*, silicate bacteria, in mobilizing potassium from certain aluminsilicates (orthoclase, microcline, mica-mucovite, and nile silt). These silicate bacteria clearly enhanced the weathering of the studied materials, mobilizing significant quantities of potassium. Due to physical and chemical degradation, the aseptic incubation of moist silicate minerals with silicate bacteria gradually increased the levels of soluble and amorphous silica. The order of release of water-soluble silica was micamuscovite, nile silt, microcline, and orthoclase. Muscovite experienced the biggest changes in soluble and amorphous aluminum content, while silt experienced the least changes. The dissolution of all the silicate minerals was positively impacted by nitrogen amendment, which indirectly increases soil fertility by raising the percentage of clay and minerals (Raturi et al. 2021; Afify 2022).

## Algae as biofertilizers

**Fig. 3:**
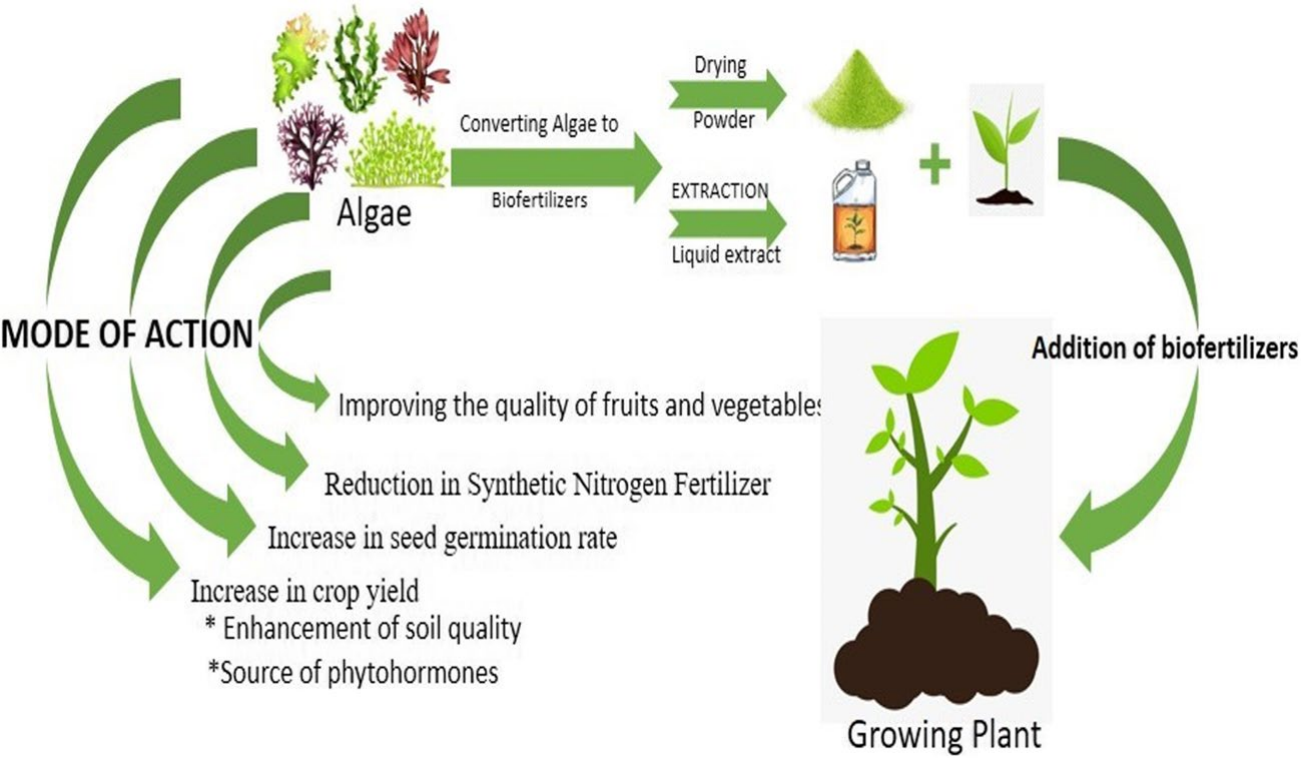
Algae as biofertilizer

The best substitutes for synthetic fertilizers are biofertilizers. Algal species hold considerable promise for biofertilizer technology in terms of affordability and environmental friendliness (Chatterjee et al. 2017). The algal pathway provides significant byproducts, and its effectiveness as a biofertilizer is its physicochemical behavior, and that soil health is enhanced. Algae are the most advantageous and in-demand bio resource of the twenty-first century due to their technological and commercial viability and environmental advantages (Mahapatra et al. 2018) (Fig. 3).

### Microalgae as biofertilizer

Eukaryotic green algae and prokaryotic blue algae are common photosynthetic microalgae. Due to their capacity to improve macro- and micronutrient consumption and enrich soil nutrients, they offer significant potential for use in modern agriculture. Microalgae can create plant growth hormones, polysaccharides, antimicrobial compounds, and other metabolites to support plant growth in addition to enhancing soil fertility and quality (Guo et al. 2020). Microalgae are an important source of biofertilizers for agriculture (Dineshkumar et al. 2019). The bioactivities and physicochemical characteristics of microalgal generated extracts (bioactive and high-value products) are used to classify them. Microalgae offer a number of advantageous qualities. Due to the inherent bioactive chemicals that increase plant productivity, they have received widespread recognition for agricultural uses (Bello et al. 2021). Microalgal biofertilizers can replace chemical fertilizers since they are easily renewable, less expensive, and can assist farmers in growing healthy organic crops and fostering an environment free of harmful chemicals (Dineshkumar et al. 2020). In place of artificial fertilizers, cyanobacteria are an effective biofertilizer that promotes plant development and crop production while improving the soil’s quality by adding organic matter to the soil (Maqubela et al. 2009). Additionally, algal biomass is a rich source of metabolites (Renuka et al. 2018; Jamal Uddin et al. 2019) (Table 3).

**Table 3:** Summary of Microalgae as biofertilizer

Microalgae	Algal strain	Synthesis of biofertilizer	Form of algal biofertilizer	Application	Statistical analysis	Mode of action	Treated plant	References
Green microalgae	*Chlorella vulgaris*	Algal culture incubated under continuous illumination 4500 l × by maintaining temperature of 25 C ± 2 C	Powder	Soil amendment	Least significant differences test at 0.05 level	Increase plant height growth, yield, biochemical and mineral components, and germinability of seeds produced	*Zea mays* L	Bulinski et al. (2012); Dineshkumar et al. (2019)
Blue-green algae	*Spirulina Platensis*	Algal culture incubated under continuous illumination 4500 l × by maintaining temperature of 35 °C ± 2 °C	Powder	Soil amendment	Least significant differences test at 0.05 level	Increase early stage growth performance, improved yield, and increased seed germination	*Zea mays* L	Bulinski et al. (2012); Dineshkumar et al. (2019)
Blue-green algae	*Spirulina platensis*	Algal culture drying, grinding, and converting to powder. Soil was treated with cow manure (10 g) and 3 g of dry powder	Powder	Soil drench	Growth estimated by graphs	Promotes leaf area, yield, and fruit quality, positive effect of soil fertility	*Allium cepa* L	Dineshkumar et al. (2020)
Green microalgae	*Chlorella vulgaris*	Algal culture drying, grinding, and converting to powder. Soil was treated with cow dung (10 g) and 3 g of dry powder	Powder	Soil drench	Growth estimated by graphs	Improves yield quantitatively and qualitatively and promotes growth	*Allium cepa* L	Dineshkumar et al. (2020)
Green microalgae	*Haematococcus pluvialis*	Algal culture pH 7.2–7.4, temperature 25 ± 2 °C for 15 days and then 50 °C for 5 days	Liquid media	Seed imbibition	Plant growth parameters assessed using ANOVA and Duncan’s multiple range test	Enhances and protects crops based on cell elements	*Phoenix dactylifera* L. (oil palm)	Sani et al. (2022)
Green microalgae	*Acutodesmus dimorphus*	Algal culture, drying, aqueous extraction	Aqueous extract	Foliar spray, seed imbibition	StatPlus:mac LE programing	Evaluate seed germination, plant growth, and fruit production	*Solanum lycopersicum* L (Tomato)	(Garcia-Gonzalez and Sommerfeld 2016; Sani et al. 2022)
Blue-green algae	*Oscillatoria* sp*.*	Algal collection then liquid extraction	Liquid extract	Foliar application	MSTAT-C computer program	Stimulates growth, and increases yield	*Abelmoschus esculentus* (L.) Moench (Okra)	(Jamal Uddin et al. 2019)
Green microalgae	*Tetraselmis* sp.	Algal cultivation, drying	Powder	Soil amendment	Microsoft Excel	Improves nutrient uptake and plant growth	*Phoenix dactylifera* L (Date palm)	(Saadaoui et al. 2019)

Algae are plentiful and easy to find in a moist environment. Algae and the substances they produce can aid in the commencement of seed germination and the growth of plant roots, which affects temperature, resilience to abiotic stress, and the capacity of plants to absorb nutrients (Zafar et al. 2022). Algal biofertilizers have potential to reduce synthetic nitrogen fertilizer use. The cyanobacteria may be able to fix up to 22.3–53.1 kg of nitrogen per hectare, which could prevent the need for 25–50% of chemical nitrogen fertilizer (Issa et al. 2014). Algal biofertilizers are able to increase seed germination rate. Winter wheat and cress seed germination were both improved by supercritical fluid extracts of spirulina biomass (Michalak et al. 2016). Also, they have the ability to increase crop yield by enhancing soil fertility, providing plant growth hormones and plant tissue colonization. They improve the quality of fruits and vegetables. Despite some challenges, algae have a promising future as biofertilizers (Table 3).

### Macroalgae as biofertilizer

The complex and widely distributed group of photosynthetic organisms known as seaweed is essential to aquatic environments (Egan et al. 2013). The aquatic plant kingdom Thallophyta includes seaweeds, commonly referred to as macroalgae, which are regarded as a vital component of the marine ecology and lives in coastal waters (Nabti et al. 2017). There are thought to be 9000 species of macroalgae. Based on the existence of photosynthetic pigment, storage capacity, and other factors, macroalgae can be divided into three primary groups based on components of food products’ cell walls and include Rhodophyta (red), Phaeophyta (brown), and Chlorophyta (green) (Khan et al. 2009; Zafar et al. 2022). Seaweeds are typically found clinging to the bottom of rather shallow coastal waters. A new generation of natural organic fertilizers called seaweed extract contains an effective nutrient source that encourages faster seed germination, increased crop yield, and increased crop resistance (Selvam and Sivakumar 2013; Suriya et al. 2018). Seaweed extracts enhance biological characteristics of the soil and raise output under biotic and abiotic stress (Yanebis Pérez Madruga 2020). Brown macroalgae, such as *Ascophyllum nodosum* (L.), are most frequently utilized in agriculture (Khan et al. 2009; Tuhy et al. 2013; Díaz-Leguizamón et al. 2016). In addition to *A. nodosum*, other brown algae such as *Ecklonia maxima* (Osbeck) *Papenfuss*, *Fucus* spp., *Laminaria* spp., *Sargassum* spp., and *Turbinaria* spp. serve as biofertilizers. Mediterranean red algae like *Corralina*, Green algae, *Pterocladia pinnata* (Hudson) Papenfuss, and *Jania rubens* (L.) J.V. such as *Enteromorpha intestinalis* (L.) *Nees*, *Cladophora dalmatica Kützing*, *Ulva lactuca* L. is also employed as a biostimulant for plant growth (Aghofack et al. 2015; Mireya Hernández-Herrera et al. 2016; Yanebis Pérez Madruga 2020). Algal extracts and inorganic (Coppens et al. 2016; Ronga et al. 2019; El-Moursy et al. 2019) (Coppens et al. 2016; Salim 2016; Wafaa et al. 2017; Ronga et al. 2019) fertilizers can be combined, which might promote sustainable agricultural productivity. They can be used in various ways, including foliar sprays (Ronga et al. 2019), soil additives (Hashem et al. 2019; Omer et al. 2019), and on seeds (Das et al. 2019; Ronga et al. 2019; Hernández-Herrera et al. 2019). Demonstrating a broad range of applications, many beneficial effects are found, such as improved germination, radicular system development, better fruit quality, increased leaf area and chlorophyll content, improved crop output, vitality, strong resilience to biotic and abiotic stress, and extended postharvest shelf-life products (Khan et al. 2009; El-Baky et al. 2010; Paudel et al. 2012; Guzmán-Murillo et al. 2013; Mireya Hernández-Herrera et al. 2016; Coppens et al. 2016; Oancea et al. 2017; Hernández-Herrera et al. 2018, 2019; Das et al. 2019; Patel et al. 2019; Sunarpi et al. 2019; Hashem et al. 2019; Ronga et al. 2019; El-Moursy et al. 2019). Macroalgae have the ability to increase crop yield, improve seed germination, improve soil characteristics, increase growth and quality of crops, and improve abiotic stress tolerance (Yanebis Pérez Madruga 2020).

Some marine macroalgae are used as biofertilizers by mixing their pulverized form with the soil. They contain unexplored reservoirs of naturally occurring physiologically active chemicals (Nabti et al. 2017). They serve as abundant sustainable botanical bioresources (Khan et al. 2009). Due to their high quantities of organic matter, which enriches soil with nutrients, marine macroalgae are effective biofertilizers (Kumareswari and Rani 2015; Layek et al. 2018). Additionally, when applied in sufficient amounts, they were superior and a more acceptable alternative to chemical and mineral fertilizers (Mirparsa et al. 2016). Marine macroalgae have numerous uses as eco-friendly fertilizers in contemporary agriculture and horticulture crop enhancement (in finely powdered form) (Hernández-Herrera et al. 2018). The application of seaweed as a soil amendment was effective on enhancing plant growth (Hernández-Herrera et al. 2014; Hashem et al. 2019). Many parts of the world employ seaweed manure to improve in agricultural soil (Eyras et al. 2013; Ramya et al. 2015) (Table 4).

**Table 4:** Marin macroalgae as biofertilizer (powder)

Macroalgae	Algal strain	Form of algal biofertilizer	Application	Mode of action	Treated plant	References
Green algae	*Ulva lactuca*	Powder	Soil amendment	Accelerates growth and alleviates the effect of high salt levels	*Brassica napus* L	Hashem et al. (2019)
Brown algae	*Cystoseira* spp.	Powder	Soil amendment	Alleviates harmful effects of salinity on canola plants and stimulates the growth and productivity	*Brassica napus* L	Hashem et al. (2019)
Red algae	*Gelidium crinale*	Powder	Soil amendment	Promotes growth hormones, improving salt stress tolerance	*Brassica napus* L	Hashem et al. (2019)
Green algae	*Halimeda microloba*	Powder	Soil drench	Increases growth and yield of plants	*Abelmoschus esculentus* (L.) Moench	Muniswami et al. (2021)
Brown algae	*Turbinaria ornata*	Powder	Soil drench	Increases number of flowers, pods, length, and weight of pods compared to foliar spray	*Abelmoschus esculentus* (L.) Moench	Muniswami et al. (2021)
Brown algae	*Sargassum* sp.	Powder	Soil drench	Increase in length and weight of pods	*Abelmoschus esculentus* (L.) Moench	Muniswami et al. (2021)

While, another marine macroalgae are used as liquid biofertilizers and are sprayed over the soil and plants. Due to their high levels of organic matter, micro and macro elements, vitamins, and other nutrients, liquid fertilizers made from natural sources, such as seaweed, are effective fertilizer alternatives for agricultural crops. Fatty acids and growth regulators are abundant (Crouch and vanStaden 1993). In agriculture and horticulture, bioactive compounds derived from marine algae are used, and numerous positive results in terms of improvement to productivity and quality have been observed (Crouch and vanStaden 1993; Blunden et al. 1996; Suriya et al. 2018). Seaweed extracts have the potential to replace chemical fertilizers with environmentally friendly liquid biofertilizers, which is essential for organic agricultural techniques leading to sustainable agriculture (Ramya et al. 2015). A variety of liquid fertilizers from seaweed are applied as a foliar spray (Thirumaran et al. 2009; Ramya et al. 2015) (Table 5).

**Table 5:** Marine macroalgae as biofertilizers (aqueous extract)

Marine macroalgae	Algal strain	Form of algal biofertilizer	Application	Mode of action	Plant	References
Brown algae	*Turbinaria ornata*	Liquid extract	Foliar application	Increase in yield, enhanced overall growth and physiology of plants	*Ocimum sanctum* Linn	Suriya et al. (2018)
Green algae	*Caulerpa racemosa*	Aqueous extract	Foliar spray	Increased in biochemical parameters in plants and enhanced overall growth	*Ocimum sanctum* Linn	Suriya et al. (2018)
Brown algae	*Sargassum wightii*	Liquid extract	Foliar application	Increased in yield and enhanced growth attributes, plant height, dry matter production, leaf area index, crop growth rate of sunflower hybrid plant	*Ocimum sanctum* Linn	Suriya et al. (2018)
Brown algae	*Stoechospermum marginatum*	Liquid extract	Foliar application	Promotes yield, biochemical, growth of plants and constituents in chlorophyll biosynthesis	*Solanum melongena* L	Ramya et al. (2015)
Brown algae	*Sargassum* sp.	Aqueous extract	Foliar spray	Enhanced yield and growth (plant height, number of branches and number of pods)	*Abelmoschus esculentus* (L.) Moench	Muniswami et al. (2021)
Brown algae	*Turbinaria ornata*	Liquid extract	Foliar spray	Increase in plant height and number of branches during early stage of growth	*Abelmoschus esculentus* (L.) Moench	Muniswami et al. (2021)
Red algae	*Laurencia obtusa*	Aqueous extract	Foliar application	Enhances seed germination, improves plant growth, and increases in potassium content	*Zea mayz* L	Safinaz and Ragaa (2013)
Red algae	*Corallina elongata*	Liquid extract	Foliar spray	Induces resistance to frost and fungal and insect attack and increases nutrient uptake from soil and plant fresh weight	*Zea mayz* L	Safinaz and Ragaa (2013)
Red algae	*Jania rubens*	Aqueous extract	Foliar application	Increases plant’s nitrogen content, plant length, and leaf number	*Zea mayz* L	Safinaz and Ragaa (2013)

## Fungi as biofertilizer

Fungi are one of the most significant taxonomic families of eukaryotic and heterotrophic living organisms on Earth and include mildew, mold, mushrooms, yeast, and puffballs They are advantageous for crop protection, plant growth, and crop yield (Arora 2019; Devi et al. 2020; Ahmad et al. 2022).

In order to enhance, add, conserve, and transform nutrients from an unusable form to a usable form, biofertilizers are made of biologically active bacterial and fungal strains (Rastegari et al. 2020a, b). Helpful fungi benefits the plant by producing siderophores, gluconase antagonists, antibiotics, and cell wall lysing enzymes like cellulases and glycosidase, among other directed multifarious plant growth-promoting characteristics. Micronutrients (phosphorus, potassium, and zinc) are also solubilized, and auxin, gibberellins, cytokinin, and ethylene are produced (Arora 2019; Abo Nouh 2019; Devi et al. 2020; Ahmad et al. 2022).

### Micro fungi as biofertilizers

Globally, the use of chemical fertilizers in agriculture has significantly increased over the past two decades, but excessive fertilizer use is having increasingly negative effects on the environment of the soil and water bodies. As a result, the idea of using mycorrhizal fungi as a biofertilizer is a promising one from the perspectives of cost effectiveness, energy conservation, and environmental friendliness (Nath Yadav and Yadav 2020; Kour et al. 2020; Thakur 2020)*.* Mycorrhiza is a massive, useful, and underutilized resource for soil ecosystem management. It is a diverse group of fungi that is mostly found on the roots of plants (Singh et al. 2019; Nath Yadav and Yadav 2020; Kour et al. 2020; Thakur 2020). Biofertilizers are broadly classified as N_2_ fixing (free-living, symbiotic, and associative symbiotic), phosphate solubilizing (bacteria and fungi), phosphate mobilizing (arbuscular mycorrhiza, ecto mycorrhiza, ericoid mycorrhizae, orchid mycorrhiza, and plant growth-promoting rhizobacteria) (Rastegari et al. 2020b). After nitrogen, phosphorus is the second-most crucial macronutrient for plants (Arora 2019; Devi et al. 2020; Ahmad et al. 2022). The amount of soluble phosphorus in soil is insufficient for plants’ metabolic processes, and a shortfall could result in slower development and decreased leaf biomass, (Arora 2019; Devi et al. 2020; Ahmad et al. 2022). Several chemical fertilizers were used to meet the shortfall but were harmful to the environment. As a result, fungi are considered as an alternative strategy, because fungi are naturally occurring organisms that provide soluble phosphorus without endangering the environment (Arora 2019; Devi et al. 2020; Ahmad et al. 2022). Plants obtain phosphorus from the earth in the form of phosphate. In comparison to other macronutrients, this element has very little mobility in the plant. Phosphorus-soluble microorganisms play an important role in phosphorus-based nutrition, increasing plant supply by releasing organic and mineral soil phosphorus pools via solvent and mineralization (Abo Nouh 2019; Aslam et al. 2022) (Table 6), while potassium is the most abundant macronutrient and is essential for plant growth and development. The most significant component of microbial communities in soil, particularly in the rhizosphere, is potassium, which is used to solubilize minerals rich in potassium. In particular, two useful communities of the arbuscular mycorrhizal fungus *G. intraradices* and *G. mosseae* could be incorporated into the soil as inoculants to promote the growth of crops (Arora 2019; Devi et al. 2020; Ahmad et al. 2022) (Table 6).

**Table 6:** Micro fungi as biofertilizers

Micro fungi	Type of biofertilizers	Group	References
*Glomus* spp.	Phosphate mobilizing biofertilizer	Arbuscular mycorrhiza	Rastegari et al. (2020a)
*Giaspora* spp.	Phosphate mobilizing biofertilizer	Arbuscular mycorrhiza	Rastegari et al. (2020a)
*Acaulospora* spp.	Phosphate mobilizing biofertilizer	Arbuscular mycorrhiza	Rastegari et al. (2020a)
*Scutellospora* spp.	Phosphate mobilizing biofertilizer	Arbuscular mycorrhiza	Rastegari et al. (2020a)
*Sclerocystis* spp.	Phosphate mobilizing biofertilizer	Arbuscular mycorrhiza	Rastegari et al. (2020a)
*Laccaria* spp.	Phosphate mobilizing biofertilizer	Ectomycorrhiza	Rastegari et al. (2020a)
*Pisolithus* spp.	Phosphate mobilizing biofertilizer	Ectomycorrhiza	Rastegari et al. (2020a)
*Boletus* spp.	Phosphate mobilizing biofertilizer	Ectomycorrhiza	Rastegari et al. (2020a)
*Amanita* spp.	Phosphate mobilizing biofertilizer	Ectomycorrhiza	Rastegari et al. (2020a)
*Pezizella ericae*	Phosphate mobilizing biofertilizer	Ericoid mycorrhiza	Rastegari et al. (2020a)
*Rhizoctonia solani*	Phosphate mobilizing biofertilizer	Orchid mycorrhiza	Rastegari et al. (2020a)
*Glomus intraradices*	Potassium solubilizing biofertilizer	Arbuscular mycorrhiza	Arora (2019); Devi et al. (2020); Ahmad et al. (2022)
*Glomus mosseae*	Potassium solubilizing biofertilizer	Arbuscular mycorrhiza	Arora (2019); Devi et al. (2020); Ahmad et al. (2022)
*Suillus bovines*	Zinc solubilization	Ericoid mycorrhiza	Arora (2019); Devi et al. (2020); Ahmad et al. (2022)
*Suillus luteus*	Zinc solubilization	Ericoid mycorrhiza	Arora (2019); Devi et al. (2020); Ahmad et al. (2022)
*Paxillus involutus*	Zinc solubilization	Ericoid mycorrhiza	Arora (2019); Devi et al. (2020); Ahmad et al. (2022)
*Oidiodendron maius*	Zinc solubilization	Ericoid mycorrhiza	Arora (2019); Devi et al. (2020); Ahmad et al. (2022)
*Hymenoscyphus ericae*	Zinc solubilization	Ericoid mycorrhiza	Arora (2019); Devi et al. (2020); Ahmad et al. (2022)
*Beauveria caledonica*	Zinc solubilization	Ericoid mycorrhiza	Arora (2019); Devi et al. (2020); Ahmad et al. (2022)

**Fig. 4:**
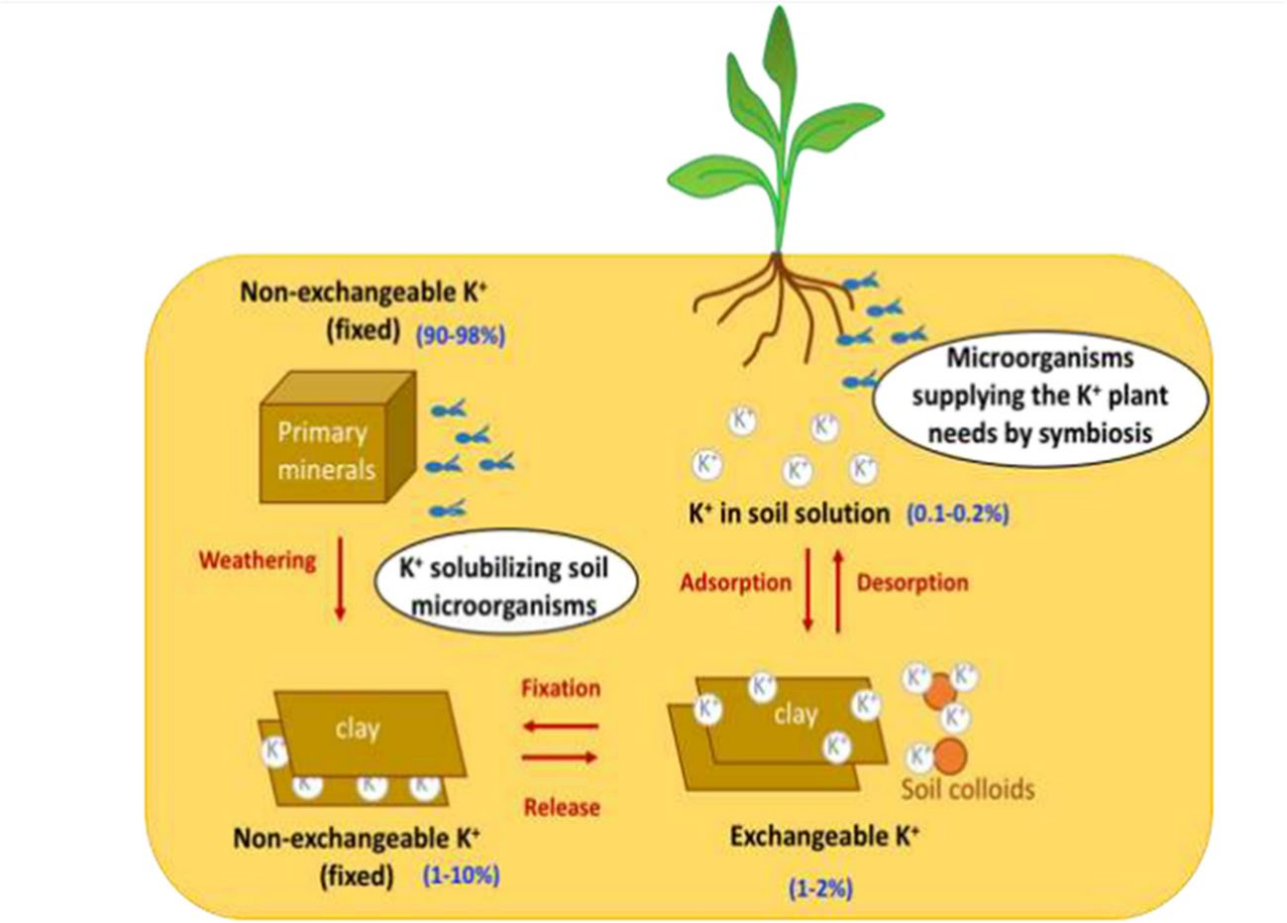
Involvement of soil-dwelling microbes in the K + cycle’s dynamics. Diagram illustrating the use of microbes to improve K + plant nutrition K + -dissolving microbes and those that interact symbiotically with plants (Copyright is access from Haro and Benito 2019)

There are five stable isotopes of zinc (Zn), which is the 23rd most plentiful element on earth (Arora 2019; Devi et al. 2020; Ahmad et al. 2022). Zinc is a key component of many metabolic processes and functions as a regulatory cofactor for enzymes and proteins. It is well known that the structural motif of the zinc finger plays a key function in the control of transcription (Arora 2019; Devi et al. 2020; Ahmad et al. 2022). Fungi create organic acids that increase the mobilization of zinc by changing its insoluble form to soluble, which is easily accessible in soil (Arora 2019; Devi et al. 2020; Ahmad et al. 2022). The potential to dissolve zinc has been seen in ericoid mycorrhiza, including *Suillus bovinus*, *Suillus luteus*, *Paxillus involutus*, *Oidiodendron maius*, *Hymenoscyphus ericae*, and *Beauveria caledonica* (Arora 2019; Devi et al. 2020; Ahmad et al. 2022) (Haro and Benito 2019) (Fig. 4, Table 6).

### Macro fungi as biofertilizers

A collection of microbiomes that interact with plant roots affects plant development and defense. Numerous bacteria, fungi, actinomycetes, and other eukaryotic microorganisms can be cultivated in microbiomes. Rhizobacteria are the bacteria that live in the rhizosphere and improve plant development and crop yield. The most essential plant growth-promoting fungi are *Penicillium*, *Trichoderma*, *Fusarium*, and *Phoma*. The cucumber has developed systemic resistance to many diseases due to the presence of many plant growth-promoting fungi species (Romera et al. 2019). These fungi are non-pathogenic saprophytes that live in soil and aid crop plants by protecting them from disease as well as boosting plant growth (Begum et al. 2019) (Table 7).

**Table 7:** Effective fungi species with their mechanisms

Fungal species	Name of mechanism	Effect on plant	References
*Aspergillus awamori* *Aspergillus niger* *Penicillium digitatum*	Phosphate solubilizing	Respiration, photosynthesis, energy transfer, signal transduction, energy accumulation, cell enlargement, cell division, and macromolecular biosynthesis	Babalola et al. (2021)
*Aspergillus sydawi* *Aspergillus tubingensis Aspergillus flavus* *Aspergillus candidus* *Aspergillus parasiticus, Aspergillus fumigatues Aspergillus flavus* *Trichoderma viride*	Organic acid production such as citric acid, gluconic acid, oxalic acid, succinic acid, malic acid, and glycolic acid	Lowering soil pH	Kalayu (2019)
*Penicillium lilacinium* *Penicillium bilaii* *Penicillium citrinum* *Penicillium balaji*	Organic acid production such as glycolic acid, oxalic acid, succinic acid, gluconic acid, malic acid, and citric acid	Lowering soil pH	Kalayu (2019)
*Aspergillus niger* *Aspergillus nomius* *Aspergillus oryzae* *Beauveria caledonica* *Penicillium luteum* *Trichoderma harzianum*	Zinc solubilizing	Improve soil fertility and crop yield	Dubey et al. (2019)
*Aphanomyces sp.* *Cylindrocladium sp.* *Fusarium sp.* *Macrophomina sp.* *Phytophthora sp.* *Pythium sp.* *Rhizoctonia sp.* *Sclerotinium sp.* *Verticillium sp.* *Thielaviopsis sp***.**	Formation of skeletal framework of macro aggregates	Aid in the uptake of Zn, Cu, Fe, Mn, and other nutrients	Dubey et al. (2019)
*Glomus intraradices* *Glomus mosseae*	Potassium solubilizing	Promotes growth of cotton, rape, pepper, cucumber, khella, sorghum, wheat, tomato, chili, Sudan grass, and tobacco	Mącik et al. (2020)

The non-pathogenic *Fusarium* species effectively promote plant growth. Competition and induction of host defenses follow two possible modes of action. There have been numerous reports of non-pathogenic *Fusarium oxysporum* strains controlling *Fusarium* wilt in various crops (El-Maraghy et al. 2020). *Phoma* sp. and *P. simplicissimum* are fungi that encourage plant growth and successfully stimulated cucumber systemic resistance to the anthracnose-causing organism *Colletotrichum orbiculare*. Results from the controlled cultivation of *Piriformospora indica* (Hymenomycetes: Basidiomycota) revealed that the species was an endophyte and stimulated plant growth (Begum et al. 2019). Vesicular–arbuscular (VA) mycorrhiza are used in biofertilizers as nutrient mobilizers and *Trichoderma* spp. are used as cellulose decomposers (Kar et al. 2021). Other species used as biofertilizers include *Aspergillus awamori*, *A. niger*, and *Penicillium digitatum*, which are phosphate solubilizers, and contribute to processes like respiration, photosynthesis, energy transfer, signal transduction, energy accumulation, cell enlargement, cell division, and macromolecular biosynthesis. Lowering soil pH through microbial generation of organic acids or the release of protons is the main mechanism for solubilizing soil phosphorus (Babalola et al. 2021). Phosphorus can precipitate in alkaline soils to create calcium phosphates, such as rock phosphate (fluorapatite and francolite), which are insoluble in the ground. As the pH of the soil lowers, their solubility rises. Phosphorus solubilizing microorganisms produce organic acids that reduce the pH of the soil, increasing the availability of phosphorus. The production of organic acids and the solubility index are strongly positively correlated. By releasing CO_2_, phosphorus solubilizing microorganisms are also known to produce acidity, as seen in the calcium phosphates’ solubility. Phosphorus was dissolved as a result of the production of organic acid and the pH drop caused by the action of microbes. Many fungi such as *Penicillium bilaii*, *Penicillium citrinum*, *Trichoderma viride*, *Aspergillus sydawi*, and *Aspergillus tubingensis* have this mechanism (Kalayu 2019) (Table 7).

Despite having an adequate total Zn concentration, insoluble Zn in the soil contributes to Zn deficiency in plants. While Zn is immobile in poorly reducing neutral or alkaline soils, it is easily transportable into plants in oxidizing acidic soil. Zn is transported in the soil solution by mass flow, diffusion, and root extension in the direction of the roots whether it is a divalent cation or complexed with ligand via several transporter systems. The active transport system, which moves Zn from the root to the shoots, is responsible for the majority of Zn absorption. The basal node retains a small amount of zinc, which controls the amount of zinc distributed throughout plants. Some fungi can solubilize insoluble Zn and include *Penicillium luteum*, *Aspergillus niger*, *A. nomius*, *A. oryzae*, *Trichoderma harzianum rifai*, and *Beauveria caledonica* (Rani et al. 2020) (Table 7).

Vesicular–arbuscular mycorrhiza interact with other microorganisms in the rhizosphere and are one of the significant soil microorganisms. Under field conditions, VA mycorrhiza inoculation significantly increased growth of transplanted chilies. Vesicular–arbuscular mycorrhiza aid in the uptake of Zn, Cu, Fe, Mn, and other nutrients by expanding the network of hyphae in their cells. By entangling soil particles and organic components to form a skeletal framework of macro aggregates, VA mycorrhiza hyphae play a significant role in soil aggregation. These aggregates improve the storage of carbon and nutrients and provide a favorable habitat for the survival and development of soil microorganisms. In organic and sustainable farming systems that rely on biological processes rather than agrochemicals to prevent plant diseases, VA mycorrhiza are particularly crucial. Plants are biologically protected by VA mycorrhiza from soil-borne diseases, including *Aphanomyces*, *Cylindrocladium*, *Fusarium*, *Macrophomina*, *Phytophthora*, *Pythium*, *Rhizoctonia*, *Sclerotinium*, *Verticillium*, and *Thielaviopsis* sp*.* (Dubey et al. 2019). A study showed that by producing organic acids including malate, citrate, and oxalate as well as protons, H^+^, and CO_2_, arbuscular mycorrhizal fungi improved the availability of K. Dual inoculation of maize with *G. intraradices* and *G. mosseae* improved K absorption, and K solubilizing microorganisms promoted the growth of cotton, rape, pepper, cucumber, khella, sorghum, wheat, tomato, chili, Sudan grass, and tobacco (Mącik et al. 2020) (Table 7).

## Plant residues as biofertilizers

Plants are like all living organisms can be used as biofertilizers by using the unused parts of them (plant residues) for manufacturing of ecofriendly biodegradable fertilizers. Plant residues are used as soil biofertilizers and include banana peels. Banana is a popular fruit, because of its taste and nutritional value. As a result, large quantities of banana peel by-products are obtained. Studies have shown that the content of bananas, such as the peel and bloom, is rich in macro- and micronutrients and promotes good health through its anti-inflammatory and anti-oxidative stress properties. Research to transform banana peels into unique new items due to their benefits like increasing soil fertility is ongoing (Kraithong and Issara 2021) (Table 8). According to the international biochar initiative (2012), banana peel biochar is a carbon-rich product that is described as “a solid compound generated through the thermo-chemical conversion of biomass in an oxygen-constrained environment” (Novak et al. 2012), (Islam et al. 2019), (Comino et al. 2020). Due to its distinctive characteristic, which enhances soil quality, banana peel biochar has recently taken center stage in research (Novak et al. 2012), (Islam et al. 2019), (Comino et al. 2020) (Table 8).

**Table 8:** Higher plants as biofertilizers

Applied plant	Biofertilizer form	Treated plant	Effect	References
Banana peel *Musa* × *paradisiaca* L	Biochar	*Ipomoea aquatica* Forssk	Increase K supplement to soil but no significant increase in plant growth	Islam et al. (2019)
	Powder	*Abelmoschus esculentus* (L.) Moench	Increase K and significant increase in height, leaf area, root length, chlorophyll content, fresh and dry weight per fruit and fruit number	el Barnossi et al. (2021)
Coffee *Coffea arabica* L	Spent coffee ground	*Brassica* sp. (cabbage, broccoli)	Increases minerals (N, K, P, Cu), seed emergence, organic matter, and soil fertility	Chrysargyris et al. (2021)
Peas (pulses food) *Lathyrus eraceus* Lam	Intercropping	Cereal crops	Replenishes soil nitrogen by fixing N from the atmosphere through nodules and symbiotic relationships with rhizobia. Pulses can disrupt disease and weed cycles linked to cereals, raise soil organic carbon and water retention, and reduce greenhouse gas emission	Powers and Thavarajah (2019)
Pomegranate peel *Punica granatum* L	Water extract	*Salvia officinalis* L	Compared to those treated with chemical fertilizers, produced greater fresh and dry mass, essential oils, suppression of free radical scavenging, carbohydrates, flavonoids, phenolic compounds, and nutritional content	Abd-Rabbu et al. (2021)

Biochar is produced using a variety of agricultural and forestry wastes, including nutshells, rice husk, pinewood, and orchard pruning biomass (Carter et al. 2013; Thornbro 2022). Banana (*Musa sapientam* L.), one of the most commercially significant fruit crops in Bangladesh, is produced at household and commercial scale in around one million tons per year (Hossain 2014). When compared to other fruit crops, it is widely consumed and accessible all year round in Bangladesh. The peel of a banana, which is regarded as waste, makes up around 18–20% of its body weight (Chenost et al. 1976) (Table 8). Banana peel waste was converted into biochar using a slow pyrolysis technique with little oxygen. Three replications of each of three rates (1, 2, and 3%) of banana peel biochar were applied to agricultural soil. K concentration in banana peel biochar was high. Compared to controls, plant production and above-ground biomass decreased in 1% banana peel biochar treatments but increased in 2 and 3% treatments. Additionally, plants cultivated with banana peel biochar were stronger, healthier, and more attractive. So, for sustainable agriculture, banana peel biochar may be an excellent source of K amendment and be used in place of chemical fertilizer as a supply of K (Islam et al. 2019) (Table 8). A popular beverage consumed worldwide is coffee (Obruca et al. 2015), and during the past several decades, wastes such as spent coffee grounds (SCG) and coffee waste, which are typically disposed of with regular trash, have expanded significantly (Mussatto et al. 2011) (Cruz et al. 2012). These are the main coffee waste by-products (45%) produced during the manufacture of instant coffee and the preparation of drinks, such as the espresso coffee extraction process (Murthy and Madhava Naidu 2012; Chrysargyris et al. 2021). Coffee grounds are used to produce biochar and as a low-cost adsorptive material for the biosorption of heavy metals (Cd, Cr, Cu, and Pb) from aqueous solutions (Kyzas 2012); (Davila-Guzman et al. 2016). The composition of SCG makes it possible to use it as soil organic amendment. Studies reveal that SCG has positive effects since it increased nitrogen, phosphorous, potassium, and organic carbon content in soils (Yamane et al. 2014; Cervera-Mata et al. 2018; Comino et al. 2020). However, huge loads of SCG deposited into landfills may contaminate water supplies, emit CO_2_, and hinder plant development (Murthy and Madhava Naidu 2012; Chrysargyris et al. 2021). Due to its poisonous nature for seedling growth, SCG is rarely used as fertilizer (Ciesielczuk et al. 2019). It is advised that SCG be combined with ash from thermal biomass treatment for golden rods (*Solidago canadensis* L.).

The effect of SCG on three brassica species, namely cauliflower F1 Skywalker (*Brassica oleracea* L. var. *botrytis*), broccoli F1 Marathon (*Brassica oleracea* L. var. *cymosa*), and cabbage F1 Paltar (*Brassica oleracea* L. var. *capitata*) was studied. It was found that SCG changed the physicochemical properties of growth media by increasing media bulk density and mineral components, which was accessible while lowering available porosity. Although stomatal conductance, one of the physiological characteristics of plants, was lowered, the SCG in the substrate had an impact on the mineral accumulation in plants, causing levels of nitrogen, potassium, phosphorus, and copper to rise (Chrysargyris et al. 2021). Spent coffee grounds have the potential to increase soil fertility, and further investigations are needed to improve the use of SCG as an amendment (Cervera-Mata et al. 2018) (Table 8).

Pomegranates are one of three fruits eaten the most over the world. Pomegranate output worldwide was predicted to be 3.8 million metric tons in 2017. Pomegranate peel accounts for 26–30% of the fruit’s weight and contains significant amounts of phenolic chemicals, such as flavonoids and hydrolyzable tannins, as well as 92% antioxidant activity (el Barnossi et al. 2021). Pomegranate fruit peel contains K, N, Ca, P, Mg, and Na, as well as B, Fe, ZN, Cu, and Mu as micronutrients (Dayarathna and Karunarathna 2021). The peels also contain amino acids, vitamins, phenolic compounds, flavonoids, anthocyanins, and tannins (Omer et al. 2019). Peel from a pomegranate is regarded as an organic fertilizer. To substitute chemical fertilizers with organic wastes, the effects of pomegranate peel formulations (powder, water extract, and ethanol extract) on the growth and chemical composition of sage were assessed. During the second harvest, 6 g/L (water extract) produced the highest fresh mass (68.5 g/plant), dry mass (18 g/plant), essential oil content (1.6%, v/w), and essential oil production (28.8 ml/100 plants) values. The peel accounted for around 500 g/kg of the overall fruit weight (Aviram et al. 2000). Pomegranate powder was effectively composted by combining, it with and without banana peels at a humidity level of 50 5%. Digestion took 15 days, and the C:N ratio dropped from 22.5 to 17. The biofertilizer created using both of these techniques enhanced germination, shoot development, root length, and leaf chlorophyll content. The biofertilizers boosted yield and phenolic acid levels in wheat grains when compared to chemical farming (Singh et al. 2013; el Barnossi et al. 2021). Thus, pomegranate peel can be considered to be an organic fertilizer (Pathak et al. 2017).

Grass pea is grown as a summer crop in Kashmir and Nepal and as a winter crop in low-lying areas, such as Bangladesh (Girma and Korbu 2012). Due to its effectiveness in fixing nitrogen, grass pea is a good green manure that increases soil fertility by adding around 67 kg/ha of additional nitrogen in a single growing season. This holds advantages for subsequent non-legume crops in terms of productivity and protein (Singh et al. 2013). Cool season legumes are the key to sustainable agriculture because they are sown in the winter, extending the growth season of cereal crops and replenishing the soil with vital nitrogen and other nutrients. Farmers in Australia observed a 30% increase in wheat production compared to mono cropped wheat after using a legume rotation (Stagnari et al. 2017). Studies from Denmark also show that during rotations with field pea and lupin, the absorption of nitrogen by a variety of crops rises by 23–59% (Stagnari et al. 2017). This legume-mediated improvement in nitrogen use efficiency offers a sustainable and cost-effective alternative to high-input fertilizer regimes because N is one of the most restricting nutrients for cereal and crop productivity. Due to pea nodules, which need P for the transformation of energy, field pea and legumes in general are problematic for sustainable agriculture since they require substantially more P input than other crops (Lambein et al. 2019) (Table 8).

Generally, pulses are highly advantageous to farming systems and have had great success in sustainable farming systems through intercropping and crop rotation with cereals. In addition to replenishing soil nitrogen through their capacity to fix N from the atmosphere via nodules and symbiotic relationships with rhizobia, pulses can disrupt disease, weed cycles linked to cereals, raising soil organic carbon, water retention and reducing greenhouse gas emission (Foyer et al. 2016; Stagnari et al. 2017; Peoples et al. 2019)(Powers and Thavarajah 2019) (Table 8).

## Future Prospects

The use of biofertilizers is unquestionably the future of agriculture where it is anticipated to take the place of chemical fertilizers. It aids the process and protects soil biodegradation performed by living things, which ultimately results in a safe technique to boost soil fertility without using chemicals residues. Additionally, we anticipate adding nanomaterials to biofertilizers could offer eco-friendly and effective substitutes. Therefore, plant diseases could be controlled and plant resistance increased to reduce environmental stress and boost plant productivity and quality.

Using simulation systems in smart agriculture for each crop and its methods like using IoT applications, Big data, and cloud computing, artificial intelligence techniques are used to study plant diversity in various regions and determine the biofertilizers needed to stimulate a species’ growth and which organisms are best at extracting this biofertilizer.

Promoting the dissemination of the culture of using biofertilizers among farmers through voluntary awareness campaigns and conducting questionnaires to assess their understanding and clarify the importance of biofertilizers over chemical fertilizers, especially in agricultural areas that are still virgin in their natural habitat, such as North Africa, East Asia, the Caribbean, and some Latin American countries through support Micro-innovative projects based on the idea of entrepreneurship to produce vital local fertilizers from their environment and support them at a lower cost for farmers commercially such as microbial biostimulants and biofilm-based biofertilizers.

## Conclusion

New environmentally friendly technologies include the use of biofertilizers that will be part of sustainable agriculture. Because of its increased nutrient content, biofertilizers enhance soil fertility and increase plant productivity. It is possible to obtain biofertilizers from a variety of organisms. These include microorganisms such as bacteria (*Azotobacter*, *Cyanobacter*, and *Nitrobacter*), microalgae (*Chlorella vulgaris*, *Spirulina platensis*, *Haematococcus pluvialis*, and *Acutodesmus dimorphus*), and micro fungi (*Glomus spp*.) Also residues of macro organisms either algae, fungi, or plants can be used as biofertilizers. In addition to their biodegradable nature, the microorganisms and residues of macro organisms can safely increase soil fertility without accumulating contaminants, which preserves the integrity of the environment and its ecosystems, so this supports the principles of sustainable development.

## Data Availability

Available via corresponding author.
